# Direction of Change in Cardiorespiratory Fitness in School-Age Children: A Longitudinal Single-Centre Study

**DOI:** 10.3390/healthcare13222871

**Published:** 2025-11-12

**Authors:** Maria Zadarko-Domaradzka, Marek Sobolewski, Emilian Zadarko

**Affiliations:** 1 Faculty of Physical Culture Sciences, College Medical, University of Rzeszów, 35-959 Rzeszów, Poland; 2 Department of Quantitative Methods, Rzeszow University of Technology, 35-959 Rzeszow, Poland; msobolew@prz.edu.pl

**Keywords:** health indices, CRF, 20mSRT, cardiovascular disease prevention

## Abstract

**Background/Objectives**: Cardiorespiratory fitness (CRF) is currently a topic of widespread interest in the field of public health, considered as the basic marker for health status assessment. Better CRF is generally accepted to be beneficial in cardiovascular and metabolic disease prevention, both in children and in adults. The aim of this study was to present the direction of change in the cardiorespiratory fitness of Polish children aged 9 to 13 in a longitudinal study. **Methods**: Three series of cardiorespiratory fitness measurements were performed on school-aged children at one-year intervals. CRF was assessed based on the number of laps run in the 20 m shuttle run test (20mSRT). In order to check the level of cardiorespiratory fitness in consecutive years, the 20mSRT results were compared to the international percentile norms, considering the children’s sex and age. **Results**: The number of completed laps shows great diversity with reference to age. Together with age, the advantage of boys in terms of the number of completed laps becomes visible. The distribution of percentile classification results in subsequent tests across the whole study population shows that a low percentage of children who were qualified for the study had their CRF below the 20th percentile, and a relatively high percentage was above the 80th percentile. **Conclusions**: For the whole test group, the results of the percentile classification did not change significantly in subsequent tests. It is worth noting, though, that together with age, CRF changes evolved towards higher values in the tested group, as a vast majority was at the level of the 50th percentile. However, still a significant group of the tested children remained within the low percentile values of CRF.

## 1. Introduction

Cardiorespiratory fitness (CRF), as one of the prognostic indicators of health and physical fitness, has become a widely spread topic of interest in the field of public health [1,2,3,4,5,6,7,8]. Research shows that a high level of CRF is closely associated with a lower risk of chronic diseases and death, both in the general and in the clinical population [9]. In 2020, having announced that only 40% of children and adolescents (aged 12–15) in the USA had, so called, healthy cardiorespiratory fitness levels, the American Heart Association appealed for more frequent monitoring of the CRF level in children and adolescents [10]. CRF has been considered to be an indicator of the current and future health status of children and adolescents [11]. A low CRF level in one’s childhood is considered a risk factor for cardiovascular diseases (CVDs). The results of studies on Spanish primary school pupils over a two-year observation period indicate that CRF levels are related to cardiovascular disease risk in children aged 6 to 10 [12]. The studies of Lin et al. (2025) indicate that a higher level of CRF is related to a decreased risk of primary hypertension in children and adolescents [6].

Physical activity and CRF are often associated with each other, yet they are distinct, albeit related, notions [10,13,14]. It is suggested that CRF can potentially increase or decrease depending on the level of physical activity [1,2].

Time trends related to cardiorespiratory fitness of children and adolescents representing 19 countries with a high or medium-high income over the years 1981–2014 show that the CRF level in children and adolescents worldwide in the years 1981–2000 dropped and then stabilised [15]. The decrease in CRF and other indicators of physical health is ascribed to various factors, including the decrease in physical activity, the increase in prevalence of sedentary lifestyle or the increase in obesity levels [10,16]. A research review shows that the time spent on high-intensity physical activity among Chinese children and adolescents is positively correlated with the maximal oxygen uptake, which indicates that the increase in the physical activity level at high intensity contributes to CRF improvement [17]. Early interventions in rhythmic physical activity in the 4–5 age group also bring measurable results in children’s motor performance [18]. It has been found that among children and adolescents, both moderate and intensive physical activity have a positive correlation with CRF, while negative correlation has been noticed between CRF and low activity [19]. A lower volume and intensity of physical activity are reflected in health indices such as BMI and CRF [20]. Meanwhile, reports from research on Polish pupils show an increase in inadequate physical activity with age, with a lack of so-called spontaneous physical activity and rare physical activity of children and adolescents with their families [21]. Only 16.8% of children and adolescents in Poland are moderately or intensively physically active for at least 60 min per day 7 days a week [22].

Research suggests that supervision and early intervention as well as preventive strategies aimed at CRF among children and adolescents can be related to maintaining health later in life [6,10,11,23].

One of the most widely spread methods of estimating CRF among children and adolescents used around the world is the 20-m shuttle run test (20mSRT) [24,25,26,27]. It is recommended that the CRF assessed by means of the 20mSRT be treated as an international surveillance measure to provide insight into the health of the population, including the paediatric population [28]. Not all researchers approach this method of CRF assessment as unequivocally as others, and some subject it to critical evaluation [29,30]. However, laboratory procedures are expensive and difficult to apply widely, which is why this method is frequently used in tests. In Poland, since the 2023/2024 school year, the 20mSRT has been an obligatory test conducted once a year in Physical Education lessons to assess the CRF of children and adolescents.

The purpose of this research was to show the direction of change in cardiorespiratory fitness of Polish school-age children in a longitudinal study.

## 2. Materials and Methods

The study was of longitudinal and single-centre character. Three series of cardiorespiratory fitness measurements as well as anthropometric and body composition measurements were performed at one-year intervals (2017, 2018 and 2019) on a group of Polish children aged 9–13 at the time of the first measurement. The children whose parents did not consent to the tests and those who did have the consent yet and were absent on the days of the tests were excluded from the study. None of the children had any medical counterindications for participation in school Physical Education classes. The age of the children was calculated based on their date of birth and the date of the test. The research procedures were carried out in compliance with the ethical standards of the Declaration of Helsinki, with the approval of the university’s bioethics committee (No. 1 June 2014) and the consent of the participants and their parents or legal guardians. The analysis covered 107 children (63 girls and 44 boys) who participated in all consecutive studies and completed the whole set of tests.

The CRF was assessed by means of the 20 m shuttle run test [31], which is a progressive aerobic exercise test. The main measure of the cardiorespiratory fitness was the number of laps of the 20mSRT run (completed) [10]. All the participants were provided identical conditions. They performed the test equipped with Polar Team2 sports testers (Polar Electro Oy, Kempele, Finland). Each child had a separate transmitter and receiver; additionally, each was assigned a guardian who supervised the correctness of the test and recorded the number of completed laps and the obtained HR_peak_ values.

The anthropometric measurements were performed in accordance with accepted procedures. Body height (BH) was measured by means of a SECA 2013 mobile stadiometer (Hamburg, Germany) with an accuracy to 1 mm, and body weight and body composition were determined through electrical bioimpedance analysis (BIA) with the use of Tanita TBF 300 scales (Japan). Additionally, BMI z-scores were calculated, and classification was performed according to World Health Organization (WHO) standards.

Additionally, basic statistical measures were calculated, including the arithmetic mean (M), standard deviation (SD), median (Me), maximum (Max) and minimum (Min) values. To assess the level of cardiorespiratory fitness in children over successive years, the 20mSRT results were referenced against international percentile norms, as used by Tomkinson et al. (2017) [24] considering sex and age specificity. The significance of changes between the measurements was evaluated by means of the Wilcoxon test. The Mann–Whitney test was used to assess the significance of differences between the change in the lap numbers over the 2-year period among the girls and the boys. Additionally, the correlation between the BMI and the change in the number of laps over the 2-year period was tested with the use of Spearman’s rank correlation coefficient. The threshold for statistical significance was set at α = 0.05. All analyses were performed using Statistica 13.3 software (TIBCO Software Inc., Palo Alto, CA, USA).

## 3. Results

### 3.1. Anthropometric Characteristics of the Test Group in Consecutive Studies

Table 1 presents the mean values and standard deviation of the height, body mass and body composition, BMI and HR_peak_ in the group of girls and boys in consecutive studies. Over the two-year observation period, it was noticed that in the group of girls, the mean changes in body height (BH) became smaller with age and in the group of boys became bigger. At each age, the mean values of body fat percentage (BF%) in the group of girls increased and in the group of boys decreased or remained unchanged. On the other hand, increases in mean fat-free mass (FFM) were observed at every age in both sexes, with these values being higher in the group of boys. A similar tendency was observed in the case of BMI. High mean values of HR_peak_ at the end of the test confirm a high level of engagement of the children during the test in consecutive studies. Detailed data are presented in Table 1.

Table 2 presents the results of BMI classification of the tested children as well as the mean values of BMI *z*-scores. Both boys and girls have a relatively high BMI; the mean *z*-score values are over 0.50, and they are higher for boys (here, the mean does not drop below 0.70). The proportion of children with overweight or obesity is also high, with a relatively higher prevalence of obesity among boys.

### 3.2. Number of Completed Laps for 20mSRT in Consecutive Studies

The main measure of cardiorespiratory fitness was the number of completed laps of the 20mSRT. Table 3 presents the mean, median and standard deviation of the number of the 20mSRT laps completed among the girls and the boys in consecutive studies (studies 1, 2 and 3). As the same people participated in consecutive studies, the results below clearly show the direction and the scale of the changes in the numbers of laps completed by the children who were one year older (study 2) and two years older (study 3) than in study 1. It is noticeable that, on average, the results of the girls underwent hardly any change, while the results obtained by the boys in study 3 were better by 12 laps than two years earlier.

In order to describe the behaviour of changes for the CRF results, for each person the difference between the results in studies 3 and 1 was calculated. Table 4 presents detailed characteristics of the distribution of CRF change between studies 1 and 3 for the groups of boys and girls. In the group of boys, over 75% obtained an increase of at least two laps over the 2-year period. Every second boy improved his result by no less than 11 laps and every fourth by over 20 laps. Among the girls, the corresponding statistical values are −6, 1 and 8. In other words, it could be said that every second girl did not actually improve her result in a significant way.

**Figure 1:**
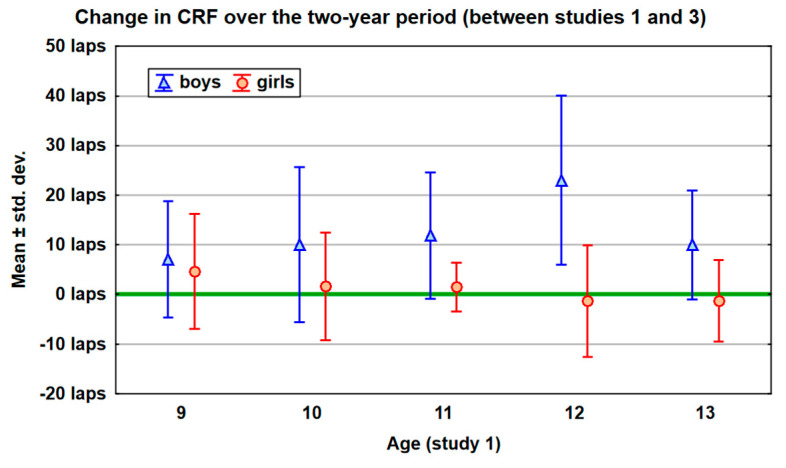
The mean change in CRF over the two-year period, with reference to the age of the children.

Figure 1 shows the mean change in CRF over the two-year period, with reference to the age of the children. It can be seen that in the group of girls, CRF grew in the periods 10–12 and 11–13; however, in older girls, it slightly dropped. In the group of boys, the increase in CRF is visible in every age group, with the largest one being in the period 12–14, approximately by over 20 laps.

### 3.3. CRF Classification According to Norms

In order to assess whether the cardiorespiratory fitness level shows improvement or decline in consecutive studies, the results were referenced to the percentile norms by Tomkinson (2017) [24], accounting for the specificity of the sex and age of the children.

The distribution of the percentile classification results in consecutive studies over the whole tested group revealed a low percentage of children with CRF below the 20th percentile and a relatively high number above the 80th percentile (42.1% in study 1; 38.3% in study 2 and 34.6% in study 3). The results of percentile classification did not change significantly in subsequent studies, which was verified by means of the Wilcoxon test (study 1 vs. 2: *p* = 0.7560; 2 vs. 3: *p* = 0.1644; 1 vs. 3: *p* = 0.3970).

The results have been grouped according to a 5-point scale and are presented in Table 5. They show the decrease in the number of children classified as ‘low’ or ‘very low’. On the other hand, the proportion of the results classified as ‘very high’ has decreased, too.

Additionally, Table 6 displays the percentage of particular CRF categories in consecutive studies, with reference to the sex of the children. The data show that boys, as compared to girls, constitute a bigger percentage of children with CRF classified as ‘very low’, and this percentage increased between the first and the third study (13.6% vs. 15.9%). In the quintile between the 20th and 40th percentiles, categorised as ‘low’, a decrease in the proportion was observed in both the boys’ and the girls’ groups. Among the boys, the proportion of people with CRF classified as ‘high’ or ‘very high’ increased. Among the girls, ‘moderate’ and ‘high’ increased, while the proportion of ‘very high’ dropped.

By means of the Wilcoxon test, the significance of changes in the CRF classification was examined, separately for boys and girls. For the group of boys, the following results were obtained: studies 1 vs. 2: *p* = 0.8415; 2 vs. 3: *p* = 0.3754; 1 vs. 3: *p* = 0.3195; for the group of girls: studies 1 vs. 2: *p* = 0.8754; 2 vs. 3: *p* = 0.7655; 1 vs. 3: *p* = 0.8845. As can be seen, neither among boys nor among girls were significant changes in the CRF classification noted between particular studies (all *p* values substantially above 0.05).

Summing up the data above the 60th percentile, it can be noted that in each consecutive study, over 50% of the participants demonstrated ‘high’ and ‘very high’ CRF, with that percentage increasing from one study to the next. In the case of boys, it was 52.3%–56.9%–61.4%, and in the case of girls, 55.5%–57.2%–58.8%.

### 3.4. BMI and CRF Changes in Consecutive Studies

In the test group, both the boys and the girls have a relative high BMI, with the percentage of people with obesity being twice as high in the group of boys as compared to girls. That is why we attempted to find a correlation between the BMI from study 1 and the change in the number of 20mSRT laps completed over the two years. In order to eliminate the age factor, BMI z-score values were analysed.

**Figure 2:**
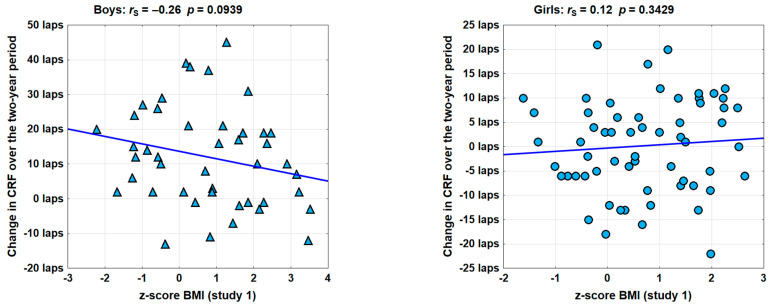
The correlation between BMI from study 1 and the two-year change in the number of the 20mSRT laps completed (*r*_S_—Spearman’s rank correlation coefficient; *p*—assessment of the significance of correlation).

Figure 2 shows the results separately for the group of girls and the group of boys. The Spearman rank correlation coefficients in the girls’ group indicate a lack of correlation between baseline BMI and the two-year change in CRF measured using the 20mSRT (*r*_S_ = 0.12; *p* > 0.10). In the boys’ group, the correlation is more visible and may be considered as close to statistically significant (*r*_S_ = −0.26; *p* < 0.10). The negative sign of the correlation coefficient indicates a smaller increase in CRF over the two-year period in boys with a higher BMI.

## 4. Discussion

As demonstrated by the preceding analyses, CRF measured by means of the 20mSRT, considering the number of laps run (completed) and referring to percentile norms, does not change significantly with age across the whole group. The results of percentile classification are not statistically significantly different between studies 1, 2 and 3. However, there is considerable individual variation, particularly in the girls’ group, where in the case of nearly half the participants, the number of completed laps drops. Also, among the boys, the change in the number of laps between studies 1 and 3 is considerably varied, though in this group, generally increases prevail. Research making use of the 20mSRT to estimate the development of CRF shows that boys improve their results in the age group 11 to 15, while the girls do not [32]. The study by Rodrigues et al., 2020, shows that children aged 6 to 15 display different patterns of CRF development, measured by means of the 20mSRT [32]. Armstrong and Welsman (2020) underline that CRF develops alongside concurrent changes in age, maturation status, morphological covariates and cardiovascular factors, with the timing and rate being specific to each individual [33].

According to our research, the number of completed laps demonstrates considerable age-related variability. With age, the advantage of boys in terms of the number of completed laps becomes apparent. The mean results of girls stay basically unchanged in subsequent studies, remaining at the level of 37–38 laps, while boys in study 3 obtain results that are better by 12 laps than two years earlier (45 vs. 57 laps).

Our observations align with those by Tomkinson et al., 2017 [24]. In their findings, boys consistently outperformed girls in each age group, obtaining better results and experiencing bigger changes with age, with the biggest increase occurring at the age of 12 [24]. In our findings also, the largest increase in CRF measured by means of the 20mSRT in the group of boys occurred in the period 12–14 years. Similar results appeared in Meng et al., 2025 [17], or in the studies by Zhang et al., 2020 [26], where boys achieved much better results in the 20mSRT in comparison with girls.

Our study demonstrated that despite the fact that girls, as compared to boys, obtained lower mean values in the 20mSRT in each consecutive study, the percentile classification showed a similar percentage of people of both sexes with CRF above the 60th percentile and a considerably lower percentage of girls with a very low CRF in comparison with boys.

There are studies covering the adult population, but also children and adolescents, that investigate the phenotype ‘fat, but fit’, showing that the increase in CRF through exercise and physical activity may counteract or mitigate the adverse effects of obesity on cardiovascular health [7,13,34,35,36]. A good CRF level may decrease the frequency of cardiometabolic disorders in children with overweight [37].

The results of a study by Sepulveda et al., 2025, indicate that children with overweight and obesity have a lower level of CRF [38]. According to Raghuveer et al., 2020, only one out of five young people with obesity has a healthy CRF [10]. The results of our studies in the group of girls did not confirm the correlation between the baseline BMI and the two-year change in CRF measured using the 20mSRT. On the other hand, in the boys’ group, this correlation was more visible, and the negative sign of the correlation coefficient indicates a smaller increase in the CRF in boys with a higher BMI over the two-year period. The studies by Dobrowolska et al., 2022, suggest that there is a correlation between body composition in children with a high BMI and the cardiopulmonary fitness level, but it is not the BMI or the body fat but the fat-free mass (FFM) that has the strongest correlation with VO_2peak_ [39]. According to Armstrong and Welsman (2020), while the 20mSRT is being performed by adolescents with overweight and obesity, metabolically neutral fat mass constitutes a burden and negatively affects performance in the test, but it does not influence the physiological variable of VO_2peak_ [33]. Raghuveer et al., 2020, point out that although it is difficult to state whether the decrease in CRF in practical tests (e.g., 20mSRT) reflects the actual worsening of the cardiorespiratory system, the tendencies in such tests more accurately reflect the direction of changes in adolescents’ typical daily aerobic activities [10].

### Limitations

A limitation of this study lies in the fact that it included exclusively the results of the 20mSRT and not physical activity or assessment of maturation, which could have explained the changes in the 20mSRT in a longitudinal observation. Considering those variables in future research might help in the better understanding of CRF. An additional limitation of the study is the fact that it used a small sample of only 107 children, which makes it impossible to generalise the results to all children. Nevertheless, a value of this study lies in its longitudinal rather than cross-sectional character. However, caution is advised when interpreting the results, and individual variability should also be considered, not solely group-level variability when the 20mSRT results are analysed by means of percentile norms [32].

## 5. Conclusions

In the 20mSRT, boys displayed much better results as compared to girls in each consecutive study. In spite of that, the whole percentile classification showed a similar percentage of people of both sexes with CRF above the 60th percentile and a considerably lower percentage of girls with a very low CRF as compared to the boys. For the whole test group, the results of the percentile classification did not change significantly over consecutive studies. It is worth noting, though, that together with age, the changes in CRF were characterised by a shift toward higher values in the studied group, with the vast majority at the level of the 50th percentile. However, still, a significant portion of the tested children remained within the low percentile values when it comes to CRF.

## Figures and Tables

**Table 1:** Mean values (standard deviation) of characteristics, body composition, BMI and HR_peak_ in consecutive studies (N = 107).

Age (Years)/ S1	*N*	BH (cm)					BW (kg)				BF (%)				BF (kg)				
		S1	S2		S3		S1	S2	S3		S1		S2	S3	S1		S2		S3
Boys (*N* = 44)																			
9	7	139.4 (3.2)	144.6 (2.9)		149.7 (4.5)		40.5 (7.9)	47.0 (11.7)	52.7 (12.4)		21.9 (8.0)		23.8 (9.6)	21.9 (8.7)	9.4 (5.0)		12.1 (7.3)		12.4 (7.7)
10	13	142.5 (6.8)	148.1 (7.9)		153.0 (7.5)		41.8 (12.7)	46.9 (15.1)	51.3 (16.0)		19.2 (8.6)		17.9 (9.7)	16.9 (10.0)	9.0 (6.5)		9.7 (7.7)		9.9 (8.4)
11	8	145.1 (5.6)	151.4 (5.8)		159.0 (5.8)		36.8 (7.6)	42.2 (9.2)	48.0 (10.3)		12.2 (5.5)		11.9 (7.7)	11.7 (8.8)	4.8 (3.0)		5.5 (4.4)		6.2 (5.8)
12	7	160.0 (9.4)	166.6 (8.2)		170.6 (5.7)		52.6 (9.6)	58.6 (11.1)	62.1 (13.8)		13.2 (7.7)		13.8 (8.2)	11.4 (6.3)	7.4 (5.8)		8.5 (6.6)		7.8 (6.9)
13	9	156.9 (7.2)	163.5 (8.4)		169.4 (6.6)		46.9 (11.0)	54.6 (13.8)	59.3 (14.3)		11.7 (9.1)		12.1 (10.4)	10.5 (8.7)	6.1 (6.4)		7.6 (8.6)		7.2 (8.5)
Girls (*N* = 63)																			
9	3	141.7 (6.1)	149.8 (4.9)		161.0 (8.2)		33.6 (5.0)	37.9 (5.9)	46.8 (5.2)		15.8 (8.2)		15.6 (8.2)	22.3 (6.2)	5.5 (3.4)		6.2 (4.0)		10.6 (4.0)
10	17	145.6 (8.2)	151.9 (8.5)		157.8 (8.2)		42.5 (10.4)	48.7 (11.9)	53.9 (12.6)		22.6 (8.4)		24.1 (7.7)	24.7 (8.9)	10.4 (6.0)		12.5 (6.5)		14.2 (7.7)
11	13	151.7 (6.3)	158.5 (6.7)		162.5 (6.1)		45.4 (9.5)	51.4 (9.4)	55.3 (9.6)		21.9 (9.4)		24.1 (9.2)	25.6 (7.5)	10.6 (6.1)		13.2 (7.0)		14.8 (6.9)
12	18	157.1 (6.2)	161.8 (5.7)		163.4 (5.3)		51.2 (8.7)	54.5 (7.6)	55.6 (8.3)		23.6 (6.8)		25.7 (6.3)	26.1 (5.3)	12.5 (5.4)		14.4 (5.2)		14.8 (5.1)
13	12	158.2 (7.4)	161.8 (6.6)		162.9 (7.5)		48.7 (7.2)	53.1 (7.3)	56.2 (7.8)		21.7 (5.9)		23.9 (4.8)	26.0 (3.9)	10.8 (4.1)		12.9 (3.9)		14.8 (4.1)
Age (years)	*N*	FFM (kg)						BMI (kg/m^2^)						HR_peak_ (bpm)					
Boys (*N* = 44)																			
9	7	31.1 (3.0)		34.9 (4.5)		40.2 (5.1)		20.9 (4.3)		22.4 (5.2)		23.5 (5.3)		200.3 (4.9)		199.9 (5.4)		200.6 (9.6)	
10	13	32.8 (6.4)		37.2 (7.8)		40.6 (8.2)		20.2 (4.6)		21.0 (5.3)		23.4 (8.9)		192.6 (15.0)		201.3 (4.9)		194.5 (8.2)	
11	8	32.0 (5.3)		36.7 (6.0)		41.8 (6.4)		17.3 (2.9)		18.3 (3.3)		19.0 (3.7)		191.3 (18.1)		200.5 (10.9)		198.1 (10.9)	
12	7	45.2 (5.6)		50.1 (7.4)		54.3 (7.1)		20.6 (3.8)		21.2 (4.3)		21.3 (4.7)		201.1 (4.0)		198.7 (3.1)		203.1 (8.3)	
13	9	40.8 (7.1)		47.0 (8.3)		52.2 (7.7)		18.9 (3.8)		20.3 (4.5)		20.6 (4.5)		193.3 (10.9)		196.8 (8.5)		196.7 (12.8)	
Girls (*N* = 63)																			
9	3	28.1 (2.6)		31.7 (2.5)		36.2 (1.3)		16.7 (1.2)		16.8 (1.6)		18.0 (0.4)		201.0 (8.9)		192.7 (11.4)		195.3 (9.3)	
10	17	32.1 (4.8)		36.2 (5.8)		39.7 (5.6)		19.8 (3.3)		20.9 (3.7)		21.5 (4.0)		199.4 (6.7)		197.6 (7.3)		195.5 (13.3)	
11	13	34.8 (4.4)		38.7 (3.9)		40.5 (3.6)		19.7 (3.9)		20.5 (3.8)		21.0 (4.2)		196.2 (13.0)		196.1 (9.3)		189.8 (14.8)	
12	18	38.6 (4.3)		40.1 (3.7)		40.7 (3.9)		20.7 (3.2)		20.8 (2.8)		20.8 (2.8)		199.0 (10.4)		195.1 (11.0)		198.6 (7.8)	
13	12	37.8 (4.0)		40.2 (4.0)		41.3 (4.0)		19.5 (3.0)		20.3 (3.1)		21.2 (3.1)		198.2 (11.1)		190.2 (13.6)		194.6 (7.8)	

S1—study 1, S2—study 2, S3—study 3, BH—body height, BW—body weight, BF—body fat, FFM—fat-free mass, BMI—body mass index, HR_peak_—heart rate peak value, and N—number of children.

**Table 2:** BMI classification of children according to WHO standards and mean BMI z-score values.

Classification BMI	Sex	S1	S2	S3
normal	boys	54.5%	51.2%	59.1%
	girls	59.0%	61.9%	66.1%
overweight	boys	20.5%	22.0%	15.9%
	girls	27.9%	25.4%	22.6%
obesity	boys	25.0%	26.8%	25.0%
	girls	13.1%	12.7%	11.3%
z-score	boys	0.79	0.81	0.74
	girls	0.69	0.63	0.52

S1—study 1, S2—study 2, S3—study 3, and BMI—body mass index.

**Table 3:** Number of completed laps for 20mSRT among girls and boys in consecutive studies.

Sex	S1			S2			S3		
	Laps 20mSRT			Laps 20mSRT			Laps 20mSRT		
	M	Me	Sd	M	Me	Sd	M	Me	Sd
boys	45.3	40.0	20.5	50.1	51.0	21.8	57.3	55.0	25.7
girls	37.9	36.0	14.0	37.2	34.0	13.6	38.2	36.0	11.5

S1—study 1, S2—study 2, S3—study 3, M—mean, Me—median, and Sd—Std. dev.

**Table 4:** Characteristics of the distribution of CRF change between studies 1 and 3 for boys and girls.

Sex	Change in CRF (Laps) Between Studies 1 and 3 (*p* < 0.001)						
	M	Me	Sd	Q1	Q3	Min	Max
Boys	12.0	11	14.2	2	20.5	−13	45
Girls	0.4	1	9.4	−6	8	−22	21

M—mean, Me—median, Sd—Std. dev., Q1—low quartile, Q3—upper quartile, Min—minimum, Max—maximum, CRF—cardiorespiratory fitness, and *p*—assessment of the significance of the difference between the two sexes in the 2-year change in the number of laps (Mann–Whitney test results).

**Table 5:** Distribution of percentile classification results in consecutive studies for the whole tested group (N = 107).

CRF Classification (Percentile Range)	S1		S2		S3	
	*N*	%	*N*	%	*N*	%
very low (poor) (<20)	7	6.5	6	5.6	7	6.5
low (fair) (20–40)	20	18.7	21	19.6	10	9.3
moderate (average) (40–60)	22	20.6	19	17.8	26	24.3
high (good) (60–80)	13	12.1	20	18.7	27	25.2
very high (very good) (>80)	45	42.1	41	38.3	37	34.6

CRF—cardiorespiratory fitness, S1—study 1, S2—study 2, and S3—study 3.

**Table 6:** The percentage distribution of individual CRF categories across successive assessments in the boys’ and girls’ groups.

CRF Classification (Percentile Range)	Boys (N = 44)			Girls (N = 63)		
	S1	S2	S3	S1	S2	S3
	%	%	%	%	%	%
very low (poor) (<20)	13.6	11.4	15.9	1.6	1.6	0.0
low (fair) (20–40)	15.9	20.5	9.1	20.6	19.0	9.5
moderate (average) (40–60)	18.2	11.4	13.6	22.2	22.2	31.7
high (good) (60–80)	15.9	20.5	20.5	9.5	17.5	28.6
very high (very good) (>80)	36.4	36.4	40.9	46.0	39.7	30.2

CRF—cardiorespiratory fitness, S1—study 1, S2—study 2, and S3—study 3.

## Data Availability

The data presented in this study are available on request from the corresponding author due to ethical restrictions related to participant confidentiality.

## Abbreviations

The following abbreviations are used in this manuscript:

20mSRT	20 m shuttle run test
CRF	Cardiorespiratory fitness
CVD	Cardiovascular disease
BMI	Body mass index
BIA	Bioelectrical impedance analysis
FFM	Fat-free mass
WHO	World Health Organization
BH	Body height
BW	Body weight
BF	Body fat
HR_peak_	Heart rate peak
VO_2peak_	Peak oxygen uptake
S1	Study 1
S2	Study 2
S3	Study 3

## References

[1] Ross R., Arena R., Myers J., Kokkinos P., Kaminsky L.A. (2024). Update to the 2016 American Heart Association Cardiorespiratory Fitness Statement. Prog. Cardiovasc. Dis..

[2] Ross R., Myers J. (2023). Cardiorespiratory Fitness and Its Place in Medicine. Rev. Cardiovasc. Med..

[3] Harber M.P., Myers J., Bonikowske A.R., Muntaner-Mas A., Molina-Garcia P., Arena R., Ortega F.B. (2024). Assessing Cardiorespiratory Fitness in Clinical and Community Settings: Lessons and Advancements in the 100th Year Anniversary of VO2max. Prog. Cardiovasc. Dis..

[4] Kaminsky L.A., Myers J., Brubaker P.H., Franklin B.A., Bonikowske A.R., German C., Arena R. (2024). 2023 Update: The Importance of Cardiorespiratory Fitness in the United States. Prog. Cardiovasc. Dis..

[5] Ruiz J.R., Cavero-Redondo I., Ortega F.B., Welk G.J., Andersen L.B., Martinez-Vizcaino V. (2016). Cardiorespiratory Fitness Cut Points to Avoid Cardiovascular Disease Risk in Children and Adolescents; What Level of Fitness Should Raise a Red Flag? A Systematic Review and Meta-Analysis. Br. J. Sports Med..

[6] Lin T., Jiang W., Lin Y., Zhang M., Zheng T., Jiang H., Liang B., Liu Y., Chen Y., Zhang Q. (2025). Association between Cardiorespiratory Fitness and Pediatric Primary Hypertension: A Case–Control Study in China. J. Hypertens..

[7] Plaza-Florido A., Altmäe S., Esteban F.J., Löf M., Radom-Aizik S., Ortega F.B. (2021). Cardiorespiratory Fitness in Children with Overweight/Obesity: Insights into the Molecular Mechanisms. Scand. J. Med. Sci. Sports.

[8] Demchenko I., Prince S.A., Merucci K., Cadenas-Sanchez C., Chaput J.-P., Fraser B.J., Manyanga T., McGrath R., Ortega F.B., Singh B. (2025). Cardiorespiratory Fitness and Health in Children and Adolescents: An Overview of Systematic Reviews with Meta-Analyses Representing over 125,000 Observations Covering 33 Health-Related Outcomes. Br. J. Sports Med..

[9] Lang J.J., Prince S.A., Merucci K., Cadenas-Sanchez C., Chaput J.-P., Fraser B.J., Manyanga T., McGrath R., Ortega F.B., Singh B. (2024). Cardiorespiratory Fitness Is a Strong and Consistent Predictor of Morbidity and Mortality among Adults: An Overview of Meta-Analyses Representing over 20.9 Million Observations from 199 Unique Cohort Studies. Br. J. Sports Med..

[10] Raghuveer G., Hartz J., Lubans D.R., Takken T., Wiltz J.L., Mietus-Snyder M., Perak A.M., Baker-Smith C., Pietris N., Edwards N.M. (2020). Cardiorespiratory Fitness in Youth: An Important Marker of Health: A Scientific Statement from the American Heart Association. Circulation.

[11] Lang J.J., Tomkinson G.R., Janssen I., Ruiz J.R., Ortega F.B., Léger L., Tremblay M.S. (2018). Making a Case for Cardiorespiratory Fitness Surveillance Among Children and Youth. Exerc. Sport Sci. Rev..

[12] Castro-Piñero J., Perez-Bey A., Segura-Jiménez V., Aparicio V.A., Gómez-Martínez S., Izquierdo-Gomez R., Marcos A., Ruiz J.R., UP&DOWN Study Group (2017). Cardiorespiratory Fitness Cutoff Points for Early Detection of Present and Future Cardiovascular Risk in Children: A 2-Year Follow-up Study. Mayo Clin. Proc..

[13] Myers J., McAuley P., Lavie C.J., Despres J.-P., Arena R., Kokkinos P. (2015). Physical Activity and Cardiorespiratory Fitness as Major Markers of Cardiovascular Risk: Their Independent and Interwoven Importance to Health Status. Prog. Cardiovasc. Dis..

[14] DeFina L.F., Haskell W.L., Willis B.L., Barlow C.E., Finley C.E., Levine B.D., Cooper K.H. (2015). Physical Activity Versus Cardiorespiratory Fitness: Two (Partly) Distinct Components of Cardiovascular Health?. Prog. Cardiovasc. Dis..

[15] Tomkinson G.R., Lang J.J., Tremblay M.S. (2019). Temporal Trends in the Cardiorespiratory Fitness of Children and Adolescents Representing 19 High-Income and Upper Middle-Income Countries between 1981 and 2014. Br. J. Sports Med..

[16] Hills A.P., Jayasinghe S., Arena R., Byrne N.M. (2024). Global Status of Cardiorespiratory Fitness and Physical Activity—Are We Improving or Getting Worse?. Prog. Cardiovasc. Dis..

[17] Meng Y., Song Y., Li H. (2025). Cardiorespiratory Fitness in Chinese Children and Adolescents: A Systematic Review and Meta-Analysis. Ann. Hum. Biol..

[18] Zhao H., Deng Y., Song G., Zhu H., Sun L., Li H., Yan Y., Liu C. (2024). Effects of 8 Weeks of Rhythmic Physical Activity on Gross Motor Movements in 4–5-Year-Olds: A Randomized Controlled Trial. J. Exerc. Sci. Fit..

[19] Jiang T., Zhao G., Fu J., Sun S., Chen R., Chen D., Hu X., Li Y., Shen F., Hong J. (2025). Relationship Between Physical Literacy and Cardiorespiratory Fitness in Children and Adolescents: A Systematic Review and Meta-Analysis. Sports Med..

[20] Kinuthia S.K., Stratton G., Wachira L.J., Okoth V.O., Owino G.E., Ochola S., Kiplamai F., Onywera V., Swindell N. (2025). Average Acceleration and Intensity Gradient of 9–11-Year-Old Rural and Urban Kenyan School-Going Children and Associations with Cardiorespiratory Fitness and BMI: The Kenya-LINX Project. PLoS ONE.

[21] Fijałkowska A., Oblacińska A., Korzycka M. (2019). Zdrowie i Styl Życia Polskich Uczniów. Raport z Badań. [Health and Lifestyle of Polish Pupils. Resarch Report.].

[22] Zembura P., Korcz A., Ciesla E., Nałecz H. (2022). Raport o Stanie Aktywnosci f Izycznej Dzieci i Młodziezy w Polsce w Ramach Projektu Global Matrix 4.0. https://www.activehealthykids.org/wp-content/uploads/2022/10/Poland-report-card-long-form-2022.pdf.

[23] Onerup A., Mehlig K., Ekblom-Bak E., Lissner L., Börjesson M., Åberg M. (2023). Cardiorespiratory Fitness and BMI Measured in Youth and 5-Year Mortality after Site-Specific Cancer Diagnoses in Men—A Population-Based Cohort Study with Register Linkage. Cancer Med..

[24] Tomkinson G.R., Lang J.J., Tremblay M.S., Dale M., LeBlanc A.G., Belanger K., Ortega F.B., Léger L. (2017). International Normative 20 m Shuttle Run Values from 1,142,026 Children and Youth Representing 50 Countries. Br. J. Sports Med..

[25] Tomkinson G.R., Carver K.D., Atkinson F., Daniell N.D., Lewis L.K., Fitzgerald J.S., Lang J.J., Ortega F.B. (2018). European Normative Values for Physical Fitness in Children and Adolescents Aged 9–17 Years: Results from 2,779,165 Eurofit Performances Representing 30 Countries. Br. J. Sports Med..

[26] Zhang F., Yin X., Bi C., Li Y., Sun Y., Zhang T., Yang X., Li M., Liu Y., Cao J. (2020). Normative Reference Values and International Comparisons for the 20-Metre Shuttle Run Test: Analysis of 69,960 Test Results among Chinese Children and Youth. J. Sports Sci. Med..

[27] Lang J.J. (2018). Exploring the Utility of Cardiorespiratory Fitness as a Population Health Surveillance Indicator for Children and Youth: An International Analysis of Results from the 20-m Shuttle Run Test. Appl. Physiol. Nutr. Metab..

[28] Tomkinson G.R., Lang J.J., Blanchard J., Léger L.A., Tremblay M.S. (2019). The 20-m Shuttle Run: Assessment and Interpretation of Data in Relation to Youth Aerobic Fitness and Health. Pediatr. Exerc. Sci..

[29] Welsman J., Armstrong N. (2021). Children’s Fitness and Health: An Epic Scandal of Poor Methodology, Inappropriate Statistics, Questionable Editorial Practices and a Generation of Misinformation. BMJ Evid.-Based Med..

[30] Armstrong N., Welsman J. (2019). Youth Cardiorespiratory Fitness: Evidence, Myths and Misconceptions. Bull. World Health Organ..

[31] Léger L.A., Mercier D., Gadoury C., Lambert J. (1988). The Multistage 20 Metre Shuttle Run Test for Aerobic Fitness. J. Sports Sci..

[32] Rodrigues L., Bezerra P., Lopes V. (2020). Developmental Pathways of Cardiorespiratory Fitness from 6 to 15 Years of Age. Eur. J. Sport Sci..

[33] Armstrong N., Welsman J. (2020). Traditional and New Perspectives on Youth Cardiorespiratory Fitness. Med. Sci. Sports Exerc..

[34] Ortega F.B., Lavie C.J., Blair S.N. (2016). Obesity and Cardiovascular Disease. Circ. Res..

[35] Eisenmann J.C., Katzmarzyk P.T., Perusse L., Tremblay A., Després J.-P., Bouchard C. (2005). Aerobic Fitness, Body Mass Index, and CVD Risk Factors among Adolescents: The Québec Family Study. Int. J. Obes..

[36] Weeldreyer N.R., De Guzman J.C., Paterson C., Allen J.D., Gaesser G.A., Angadi S.S. (2025). Cardiorespiratory Fitness, Body Mass Index and Mortality: A Systematic Review and Meta-Analysis. Br. J. Sports Med..

[37] Pires R.C., Martins H.X., Barbosa M., Molina M.d.C.B. (2025). Association of the Combination of Corporal Adiposity and Cardiorespiratory Fitness with Cardiometabolic Risk Factors in Children—PREVOI Study. Rev. Paul. Pediatr..

[38] Sepúlveda C., Monsalves-Álvarez M., Troncoso R., Weisstaub G. (2025). Children and Adolescents with Overweight or Obesity Exhibit Poor Cardiorespiratory Performance and Elevated Energy Expenditure during an Exercise Task. PLoS ONE.

[39] Dobrowolska A., Domagalska-Szopa M., Siwiec A., Szopa A. (2022). Association between Cardiopulmonary Capacity and Body Mass Composition in Children and Adolescents with High Body Weight: A Cross-Sectional Study. Children.

